# Effects of a Muay Thai Championship on Neuromuscular Parameters and Their Relationship with Competitive Outcome: A Pilot Study

**DOI:** 10.3390/jfmk11020142

**Published:** 2026-03-31

**Authors:** Iván Sotelo-Besada, Sergio López-García, Pelayo Diez-Fernández, Brais Ruibal-Lista

**Affiliations:** 1 Facultad de Ciencias de la Educación y del Deporte, Universidad Rey Juan Carlos, 28933 Madrid, Spain; ivsotelobesada@gmail.com; 2 Facultad de Educación, Universidad Pontificia de Salamanca, 37007 Salamanca, Spain; slopezga@upsa.es; 3 Grupo de Investigación en Deporte, Actividad Física y Salud (GIADES), Universidad Pontificia de Salamanca, 37007 Salamanca, Spain; brais.ruibal@frayluis.com; 4 Departamento Biología Funcional, Facultad de Medicina y Ciencias de la Salud, Universidad de Oviedo, 33006 Oviedo, Spain; 5 Escuela Universitaria de Magisterio “Fray Luis de León”, Universidad Católica de Ávila, 47010 Valladolid, Spain

**Keywords:** Muay Thai, physical performance, fatigue, vertical jump, explosive strength

## Abstract

**Background**: This study aimed to analyze the acute effects of an official Muay Thai championship on neuromuscular performance, assessing changes in upper- and lower-limb power before and after competition, as well as differences according to competitive outcome (winners vs. losers). **Methods**: Thirty-three amateur Muay Thai athletes (17 men and 16 women) performed a countermovement jump (CMJ) and a reactive push-up test (RPU) immediately before and after their bouts. Neuromuscular performance changes were analyzed using mixed-design ANOVA, and a binary logistic regression was conducted to examine the association between post-competition performance and match outcome. **Results**: Significant post-competition declines were observed in both CMJ and RPU performance (*p* < 0.001), indicating acute neuromuscular fatigue. Men exhibited higher performance values than women in both tests, with sex-specific differences in CMJ fatigue magnitude. When analyzed by competitive outcome, losers showed substantially greater performance decrements than winners, particularly in CMJ. Logistic regression revealed that post-competition CMJ performance was a significant predictor of winning, with higher CMJ values associated with greater odds of competitive success. **Conclusions**: An official Muay Thai bout induces marked acute neuromuscular fatigue affecting both upper and lower limbs, with greater impairments observed in non-winning athletes. The ability to maintain lower-limb explosive power under fatigue appears to be associated with competitive success. Simple neuromuscular assessments such as CMJ and RPU may be useful tools for monitoring fatigue, guiding tactical decisions, and informing post-competition recovery strategies in Muay Thai athletes.

## 1. Introduction

Combat and opponent-based sports are characterized by an intermittent structure, with alternating periods of high-intensity effort and partial recovery [1], which decisively influences the onset and manifestation of fatigue during competition [2]. In this context, fatigue is not inherent to the sport itself but arises as a consequence of the effort dynamics, the frequency of explosive actions, the body segments involved, and the specific moment within the bout in which these actions occur.

Available evidence in striking combat sports indicates that official competition induces acute fatigue accompanied by neuromuscular and functional alterations immediately post-bout [3], as well as impairments in motor performance under conditions of acute fatigue in contact sport athletes [4].

Within this group, Muay Thai exhibits distinctive technical–tactical characteristics that differentiate it from other combat sports such as Kickboxing, including the combined use of punches, kicks, knees, and elbows, as well as clinch fighting. These characteristics impose specific neuromuscular demands and may lead to different fatigue profiles [5]. Therefore, extrapolation of findings obtained from other combat sports should be approached with caution. Furthermore, evidence suggests that neuromuscular and fatigue responses may differ between males and females, highlighting the need to examine these effects in well-characterized, sport-specific samples [6].

Although previous studies have not found significant differences between winners and losers in physiological or hormonal variables [7,8], evidence shows that winners tend to adopt a more offensive and effective style, characterized by a higher number of head-directed techniques, counterattacks, and roundhouse kicks; patterns that may be associated with a greater ability to maintain neuromuscular performance under fatigue [9,10].

Despite extensive examination of physical and physiological aspects in combat sports such as Kickboxing [8,11,12,13], evidence specifically focused on neuromuscular variables in Muay Thai particularly when assessed in real competitive settings remains limited. In this regard, Cimadoro et al. [12] demonstrated that different frequencies of roundhouse kicks induce acute neuromuscular fatigue measurable via the countermovement jump (CMJ), highlighting this test as a sensitive tool for assessing fatigue in combat sport athletes.

However, very few studies have evaluated acute neuromuscular fatigue using CMJ and reactive push-ups (RPU) in real Muay Thai bouts, and even fewer have explored their direct relationship with fight outcome (winning or losing). This limits understanding of how the ability to maintain neuromuscular performance under fatigue may influence competitive performance.

The CMJ is widely used as a non-invasive, sensitive, and reliable measure of neuromuscular fatigue and lower-limb explosive power in high-performance sports [14]. Similarly, plyometric push tests, such as the reactive push-up, have proven to be reproducible assessments of upper-limb explosive power and, therefore, appropriate tools to detect neuromuscular alterations induced by intense exercise [15].

Accordingly, the aim of the present study was to analyze the effects of an official Muay Thai championship on neuromuscular variables, evaluating changes in upper- and lower-limb power before and after competition, as well as comparing these responses between winners and losers. It was hypothesized that bouts would induce a significant reduction in neuromuscular power in both the upper and lower limbs, and that these alterations would be associated with competitive outcome.

## 2. Materials and Methods

### 2.1. Study Design

This study employed an observational, longitudinal, and prospective design, with repeated inter- and intra-subject measurements taken before and after an official Muay Thai bout. The research was conducted in a real competitive environment, providing high ecological validity and a representative context for capturing motor and neuromuscular responses aligned with the actual demands of competition [16].

Given the limited sample size and the uneven sex distribution, this study should be considered exploratory in nature. The primary aim was to provide preliminary insights into the acute neuromuscular responses induced by real Muay Thai competition under ecologically valid conditions, thereby generating hypotheses and methodological foundations for future studies with larger and more balanced samples.

### 2.2. Participants

A total of 40 amateur Muay Thai athletes (20 men and 20 women) were invited to participate in the study. Of these, 33 athletes (17 men and 16 women) completed all testing procedures and were included in the final analysis (age: 24.7 ± 6.5 years; height: 167 ± 1 cm; body mass: 61.0 ± 10.0 kg; body mass index: 21.9 ± 3.4).

The sample was selected through non-probability convenience sampling and included athletes officially registered in the championship who voluntarily agreed to participate. All participants were assessed uniformly before and after their bout, with no manipulation of variables or assignment to conditions.

Participant recruitment began one week prior to the event through an informational circular distributed to clubs and was completed on the day of the championship through direct contact with coaches and athletes. Inclusion criteria were: official registration in the championship, absence of disabling prior injuries or medical conditions limiting performance, and full completion of the evaluation protocol (pre- and post-bout). Twelve participants were excluded due to incomplete post-bout assessments or injuries sustained during the bout.

### 2.3. Procedure

One week before the championship, an informational circular was sent to the participating clubs describing the study’s objectives, conditions, and protocol. Final recruitment occurred on the day of the event through a formal presentation of the project by the research team to coaches and athletes.

Verbal and written information about the study—including its aims, potential benefits, and risks—was provided. Age-appropriate informed consent forms were then distributed, and only athletes who signed the consent were included.

All athletes participated in a single official bout conducted under national federation rules, consisting of 3 rounds of 3 min each, with 2-min rest intervals between rounds.

Data collection was carried out by the research team with supervision from the championship’s technical and medical personnel and consisted of two phases:

1. Pre-bout phase: recording of personal data (age, weight, height, sex) and administration of the Countermovement Jump (CMJ) and Reactive Push-Up Test (RPU).
2. Post-bout phase: repetition of the same physical tests, provided the athlete’s condition allowed it, following predetermined safety criteria. The match result (win or loss) was also recorded.

Pre-competition assessments were conducted 5–8 min before the start of the bout, following the standardized warm-up routine and post-competition measurements were performed within 5–8 min after bout completion. The time interval between bout completion and post-testing was consistent across all athletes. The order of tests was standardized for all participants (CMJ followed by RPU assessment).

The method of bout termination (knockout, technical knockout, or points decision) was recorded from official competition reports. Due to the limited number of athletes within each termination category, this variable was not included in the inferential analysis.

### 2.4. Tests and Instruments

The Countermovement Jump (CMJ) and Reactive Push-Up (RPU) tests were selected due to their sensitivity to acute neuromuscular fatigue and their practical applicability in combat sports. Given that Muay Thai performance depends on explosive force production in both lower and upper limbs, these tests provide a valid assessment of neuromuscular function in key anatomical segments involved in kicking and punching actions.

To assess physical performance, the following tests were administered:

(A) Countermovement Jump Test (CMJ):

Used to assess vertical jump capacity as an indicator of lower-limb explosive power. The test was performed on a contact platform (Chronojump Boscosystem 2.3.0), recording jump height in centimeters.

Participants began in an upright position with hands on hips, performed a rapid countermovement descent, and executed a maximal vertical jump without arm swing. Three trials were completed, with 90 s of rest between attempts, and the highest value was recorded. The test has demonstrated high reliability and validity [17,18,19,20].

(B) Reactive Push-Up Test (RPU):

Used to measure reactive upper-limb pushing force. The test was performed on a contact platform (Chronojump Boscosystem 2.3.0), recording jump height in centimeters.

Participants were instructed to perform the technique as follows: begin in a push-up position with hands on the platform, maintain a straight trunk throughout the movement, jump and land on the platform, and initiate the movement with a rapid and explosive countermovement involving elbow, shoulder, and wrist flexion–extension. The test has demonstrated test–retest reliability in athletes [21,22] and utility in sport contexts [23,24].

### 2.5. Statistical Analysis

Statistical analyses were conducted using SPSS software (SPSS v.27, IBM Corporation, New York, NY, USA).

Results for each variable were expressed as absolute and relative frequencies (percentages) or as measures of central tendency and dispersion (mean and standard deviation), depending on the type of variable. Differences between groups and sex were analyzed using independent-samples Student’s *t*-tests or Mann–Whitney U tests, depending on data normality.

Differences between pre- and post-competition values were analyzed using paired-samples Student’s *t*-tests or Wilcoxon signed-rank tests according to data normality, which was assessed using the Shapiro–Wilk test. Statistical significance was set at *p* < 0.05.

Effect sizes were calculated for all comparisons. Cohen’s d was used for parametric tests, and the r coefficient derived from the z value was used for non-parametric tests. Values were interpreted as small (d ≈ 0.2; r ≈ 0.1), medium (d ≈ 0.5; r ≈ 0.3), and large (d ≥ 0.8; r ≥ 0.5).

A binary logistic regression analysis was performed to examine the association between post-test performance variables and match outcome. The dependent variable was match outcome (coded as 1 = Winners and 0 = Losers). The independent variables entered into the model were post-test countermovement jump performance (CMJPost), post-test reactive push-up performance (RPUPost), sex, and age.

Pearson’s correlation coefficient (R) was used to assess the relationships between performance variables. Correlation coefficients are reported as R values, and statistical significance was set at *p* < 0.05.

### 2.6. Ethical Considerations

**Figure 1:**
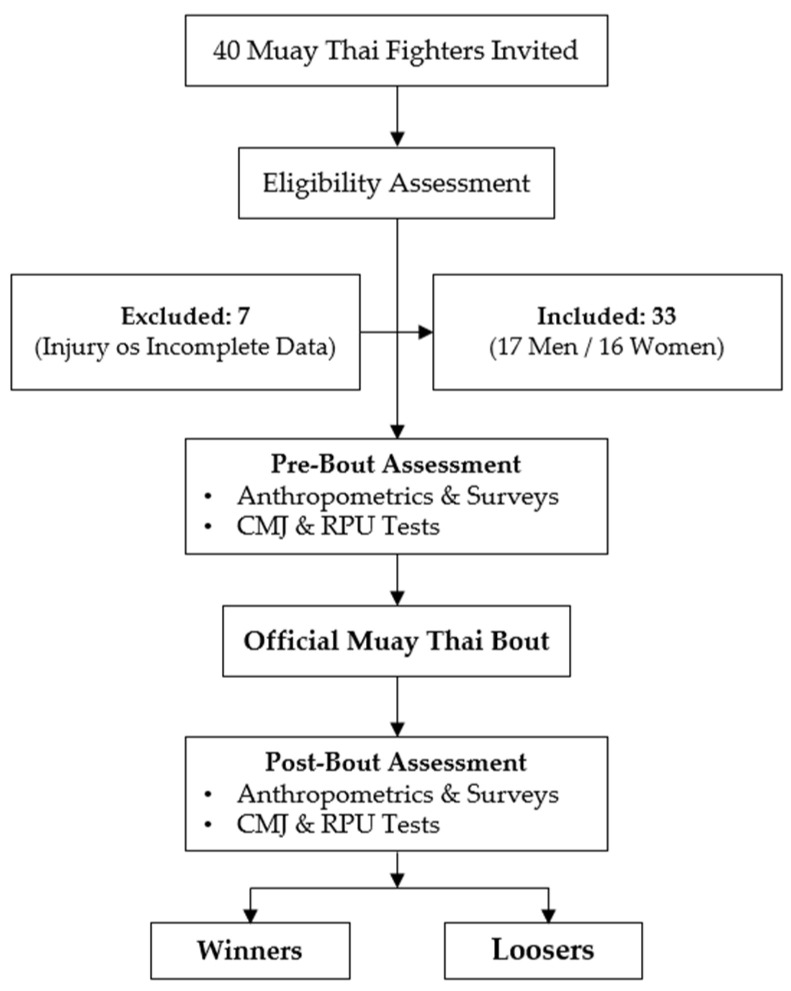
Flowchart of the study design, participant recruitment, and data collection procedures.

All participants (or their legal guardians, in the case of minors) signed an informed consent form prior to inclusion in the study (Figure 1). The protocol was approved by the Ethics Committee of Rey Juan Carlos University (internal registration number: 130420254052025) and complied with the principles of the Declaration of Helsinki [25].

## 3. Results

### 3.1. Comparison Between Pretest and Posttest in CMJ and RPU: Men vs. Women

Table 1 presents the results of the mixed-design ANOVA examining changes in CMJ and RPU performance between pre-test and post-test, with sex (Men vs. Women) as the between-subject factor and time (pre- vs. post-competition) as the within-subject factor.

For CMJ performance, a significant main effect of time was observed (F = 16.340, *p* < 0.001, η^2^_p_ = 0.345), indicating a general decrease in jump performance following the competition, with higher values consistently recorded in the pre-test. A significant main effect of sex was also found (F = 15.000, *p* < 0.001, η^2^_p_ = 0.326), with men exhibiting higher CMJ values overall than women. Additionally, the time × sex interaction was significant (F = 4.997, *p* = 0.033, η^2^_p_ = 0.139), suggesting that the magnitude of performance decline in CMJ differed between men and women. Pairwise comparisons confirmed that, regardless of statistical significance, pre-test values were always higher than post-test values.

Regarding RPU performance, a significant main effect of time was detected (F = 13.490, *p* < 0.001, η^2^_p_ = 0.303), reflecting a general decrease after the competition, with pre-test values consistently exceeding post-test values. The main effect of sex was also significant (F = 34.322, *p* < 0.001, η^2^_p_ = 0.525), showing that men achieved a higher mean jump height than women overall. The time × sex interaction was not significant (F = 0.005, *p* = 0.942, η^2^_p_ = 0.000), indicating that the decrease in RPU performance over time was similar for both sexes.

Overall, the partial eta squared values indicate medium to large effect sizes, highlighting the substantial negative impact of the Muay Thai competition on neuromuscular performance, with sex-specific differences observed for CMJ but comparable declines in RPU across men and women.

When analyses were conducted according to the sex-based, men obtained CMJ values of 32.2 ± 4.9 vs. 30.9 ± 6.1 cm (*p* = 0.138) and RPU values of 11.4 ± 3.8 vs. 10.3 ± 4.2 cm (*p* = 0.036; d = 0.53). In contrast, women obtained CMJ values of 27.8 ± 2.9 vs. 23.3 ± 4.6 cm (*p* = 0.002; d = 0.95) and RPU values of 5.5 ± 1.3 vs. 4.1 ± 1.5 cm (*p* = 0.002; d = 0.98).

### 3.2. Comparison Between Pretest and Posttest in CMJ and RPU: Winners vs. Losers

Table 2 presents the results of the mixed-design ANOVA conducted to analyze the effect of a Muay Thai competition on performance in the countermovement jump (CMJ) and the reactive push-up test (RPU), considering time (pre-competition vs. post-competition) as the within-subject factor and competition outcome (winners vs. losers) as the between-subject factor.

For CMJ performance, a significant main effect of time was observed (F = 71.032, *p* < 0.001, η^2^_p_ = 0.696), indicating a significant reduction in jump performance following the competition, with consistently higher values recorded in the pre-test. A significant main effect of group was also found (F = 12.976, *p* = 0.001, η^2^_p_ = 0.283), revealing overall differences in CMJ performance between winners and losers when averaged across time points. Furthermore, the time × group interaction was significant (F = 90.118, *p* < 0.001, η^2^_p_ = 0.744), suggesting that the magnitude of the competition-induced decline in CMJ performance differed between winners and losers.

Regarding RPU performance, the analysis revealed a significant main effect of time (F = 29.514, *p* < 0.001, η^2^_p_ = 0.710), reflecting a significant decrease in performance from pre- to post-competition, with superior pre-test values in all pairwise comparisons that reached statistical significance. In contrast, the main effect of group was not statistically significant (F = 3.066, *p* = 0.090, η^2^_p_ = 0.090), indicating no overall differences in RPU performance between winners and losers when time was not considered. However, a significant time × group interaction was detected (F = 91.517, *p* < 0.001, η^2^_p_ = 0.748), demonstrating that the extent of post-competition performance decline differed according to competition outcome.

Overall, the large partial eta squared values indicate very large effect sizes, highlighting the substantial negative impact of a Muay Thai competition on neuromuscular performance, with differential fatigue responses between winners and losers.

Pairwise comparisons showed that CMJ performance significantly decreased from pre- to post-competition, with values of 30.2 ± 4.9 cm vs. 27.4 ± 5.9 cm (*p* < 0.001; d = 0.63). Similarly, RPU performance decreased from 8.6 ± 4.3 to 7.4 ± 1.5 cm (*p* < 0.001; d = 0.65).

When analyses were conducted according to competition outcome (Winners vs. Losers), no significant differences were observed in CMJ pre-competition (31.0 ± 4.9 vs. 29.1 ± 4.1 cm; *p* = 0.222). However, significant differences emerged in CMJ post-competition (31.5 ± 5.2 vs. 21.9 ± 4.0 cm; *p* < 0.001; d = 2.01). Similarly, RPU pre-competition values did not differ significantly between winners and losers (9.0 ± 4.5 vs. 7.9 ± 3.9 cm; *p* = 0.480), whereas RPU post-competition values were significantly higher in winners compared to losers (9.2 ± 4.7 vs. 5.1 ± 2.9 cm; *p* = 0.008; d = 0.99).

### 3.3. Binary Logistic Regression Analysis

A binary logistic regression analysis was conducted to examine whether post-competition CMJ performance (CMJPOST) predicted competitive outcome (0 = Loser, 1 = Winner). A total of 33 participants were included, with no missing data.

The Omnibus test of model coefficients indicated that the model significantly improved prediction compared to the null model, χ^2^(1) = 22.982, *p* < 0.001. The Hosmer–Lemeshow goodness-of-fit test was non-significant, χ^2^(8) = 4.747, *p* = 0.784, indicating no statistically significant differences between observed and predicted values and therefore an adequate model fit.

The model explained a substantial proportion of variance in competition outcome (Cox & Snell R^2^ = 0.502; Nagelkerke R^2^ = 0.674), indicating moderate-to-high explanatory power.

CMJPOST emerged as a statistically significant predictor of winning (B = 0.443, SE = 0.144, Wald = 9.453, *p* = 0.002). The corresponding odds ratio (Exp(B) = 1.557) indicates that for each 1 cm increase in post-fight CMJ height, the odds of winning increased by 55.7%. Conversely, for each 1 cm decrease in CMJPOST, the odds of winning decrease by approximately 35.8% (1 − 1/1.557). The 95% confidence interval for the odds ratio [1.174–2.065] does not include 1, further supporting the statistical significance and robustness of the predictor.

The classification table showed an overall accuracy of 81.8%, with 84.2% of winners and 78.6% of losers correctly classified, representing a substantial improvement over the null model (57.6%). Overall, post-competition CMJ performance was a significant and meaningful predictor of competitive success. Higher CMJPOST values were associated with a greater probability of winning, and the model demonstrated adequate fit, strong discrimination ability, and stable parameter estimates.

## 4. Discussion

The present study examined the acute effects of an official Muay Thai bout on neuromuscular performance, as assessed through the Countermovement Jump (CMJ) and the Reactive Push-Up Test (RPU). The main findings indicate a statistically significant post-competition reduction in performance in both tests, reflecting the presence of acute neuromuscular fatigue induced by the physical demands of the bout. Notably, these decrements were more pronounced in non-winning athletes, particularly for CMJ performance, suggesting a greater impairment in lower-limb explosive capacity among those who were unable to achieve a competitive victory.

These results are consistent with previous investigations examining neuromuscular fatigue in combat sports. Specifically, studies analyzing repeated kicking actions in Muay Thai have reported immediate reductions in CMJ height following high-intensity striking sequences, supporting the sensitivity of this test to detect acute fatigue responses [12,26]. Performance decrements were especially evident under conditions characterized by intense yet intermittently spaced actions, reinforcing the notion that fatigue can manifest rapidly following sport-specific efforts. Collectively, these findings corroborate the use of CMJ as a reliable indicator of neuromuscular fatigue in striking-based combat sports.

Regarding upper-limb performance, although no studies have specifically evaluated RPU responses in Muay Thai athletes, CMJ has been widely validated as a marker of both central and peripheral neuromuscular fatigue across endurance and intermittent sports [27,28,29]. Given that the RPU similarly relies on the stretch–shortening cycle and rapid force production, it is reasonable to infer that explosive upper-body performance is also negatively affected by competition-induced fatigue. Interestingly, unlike the CMJ, RPU performance did not show significant differences between winners and losers. This might suggest that upper-body fatigue is less discriminatory regarding the match outcome, perhaps due to the dominant role of the lower body in Muay Thai scoring (e.g., kicks and knees) or the specific technical–tactical nature of the test compared to the clinch and striking demands of the bout.

While acute neuromuscular fatigue appears to be a common response across combat sports, its magnitude and expression may vary according to the specific technical–tactical demands of each discipline. Reductions in CMJ performance comparable to those observed in the present study have been reported in kickboxing, taekwondo, and boxing following simulated or real competition, suggesting a shared fatigue profile associated with intermittent high-intensity striking actions and repeated accelerations and decelerations [12,26]. These similarities highlight a common physiological underpinning across striking-based combat sports.

Nevertheless, Muay Thai exhibits distinctive characteristics that may exacerbate or alter neuromuscular fatigue patterns compared with other combat disciplines. The frequent use of knees and elbows, prolonged clinch exchanges, and sustained close-range combat impose substantial isometric and dynamic demands on both the upper and lower limbs, as well as on trunk musculature. These unique elements may contribute to a more pronounced and globally distributed neuromuscular fatigue response, particularly affecting lower-limb explosive performance, as evidenced by the larger CMJ decrements observed in the present study. Consequently, although Muay Thai shares general fatigue mechanisms with other combat sports, its specific technical and physiological demands likely produce sport-specific neuromuscular outcomes that warrant targeted investigation.

With respect to sex differences, studies on peripheral fatigue have observed that men show greater reductions in isometric strength after exertion compared to women, revealing sex-specific patterns of neuromuscular fatigue [30]. In the present study, the non-significant findings (*p* = 0.144 for CMJ; *p* = 0.103 for RPU) preclude any definitive explanation regarding sex-specific responses. However, it could be hypothesized for future research with adequate sample sizes that factors such as lower competitive experience or differences in muscle mass in some female athletes might influence neuromuscular fatigue patterns.

Binary logistic regression analysis revealed that better post-fight CMJ performance was associated with a higher likelihood of winning. This finding supports the idea that maintaining neuromuscular performance under fatigue is relevant in combat sports. Although no exact studies have explored this, authors such as Cimadoro et al. [12] suggest that the ability to maintain explosive power under fatigue discriminates performance levels in contact sports.

Moreover, the application of this study in a real competitive environment reinforces its ecological validity and practical relevance. Meta-analytic studies on fatigue monitoring via CMJ highlight that its use in real-world contexts provides greater sensitivity for detecting acute changes in neuromuscular status [30,31].

## 5. Limitations

Several limitations of the present study should be acknowledged. First, the relatively small sample size means that these findings should be interpreted with caution. Accordingly, this study should be considered exploratory or pilot in nature. Although differences between men and women were observed in CMJ and RPU performance, these comparisons did not reach statistical significance. Given the small sample size, these results should be interpreted with caution and considered preliminary. Future research should employ longitudinal designs with larger samples to better characterize neuromuscular fatigue responses across sexes, competitive levels, and repeated bouts. 

Regarding the potential influence of weight categories, this variable was neither measured nor controlled in the present study. Therefore, its possible effect on the analyzed variables cannot be ruled out and should be acknowledged as a limitation. Future research should consider controlling for or specifically analyzing weight categories in order to determine their potential impact on the results. Additionally, integrating complementary measures such as kinematic, physiological, or perceptual variables could provide a more comprehensive understanding of fatigue mechanisms in Muay Thai competition.

## 6. Conclusions

From a practical standpoint, these findings offer several relevant implications for coaches and strength and conditioning professionals working with Muay Thai athletes. First, neuromuscular fatigue monitoring can be efficiently implemented using simple and time-effective tests such as the CMJ and RPU, both before and after competition or high-intensity training sessions, to inform individualized recovery strategies.

Second, given the association between post-bout CMJ performance and competitive success, integrating training drills that challenge explosive power under fatigued conditions may enhance athletes’ capacity to maintain performance throughout the bout.

The observed post-bout reductions in CMJ performance highlight the importance of recovery strategies, particularly in tournament formats where athletes may be required to compete multiple times within a short period. Differences in neuromuscular recovery capacity could partially explain the competitive advantage observed in winners.

## Figures and Tables

**Table 1:** Comparison of CMJ and RPU performance between pre-test and post-test: Men and Women.

Variables	Effect	gl	F	*p*	η^2^_p_	Interpretation
CMJ	Test (Pre-Post)	1	16.340	<0.001	0.345	Significant differences
	Sex (Men vs. Women)	1	15.000	<0.001	0.326	Significant differences
	Test × Sex	1	4.997	0.033	0.139	Significant differences
RPU	Test (Pre-Post)	1	13.490	<0.001	0.303	Significant differences
	Sex (Men vs. Women)	1	34.322	<0.001	0.525	Significant differences
	Test × Sex	1	0.005	0.942	0.000	No Significant differences

CMJ: Countermovement Jump; RPU: Reactive Push-Up Test; gl: Degrees of Freedom; F: F-value (F-statistic); *p*: *p*-value (probability value); η^2^_p_: Partial Eta Squared (partial eta squared effect size).

**Table 2:** Comparison of CMJ and RPU performance between pre-test and post-test: Winners and Losers.

Variables	Effect	gl	F	*p*	η^2^_p_	Interpretation
CMJ	Test (Pre–Post)	1	71.032	<0.001	0.696	Significant differences
	Group (W-L)	1	12.967	0.001	0.283	Significant differences
	Test × Group	1	90.118	<0.001	0.744	Significant differences
RPU	Test (Pre–Post)	1	29.514	<0.001	0.710	Significant differences
	Group (W-L)	1	3.066	0.090	0.090	No Significant differences
	Test × Group	1	91.517	<0.001	0.748	Significant differences

CMJ: Countermovement Jump; RPU: Reactive Push-Up Test; gl: Degrees of Freedom; F: F-value (F-statistic); *p*: *p*-value (probability value); η^2^_p_: Partial Eta Squared (partial eta squared effect size).

## Data Availability

The data that supports the findings of this study are available from the corresponding author, I.S.-B., upon reasonable request. The data is not publicly available due to privacy and ethical restrictions.

## References

[1] Ružbarský P., Němá K., Perič T., Ambroży T., Bąk R., Niewczas M., Rydzik Ł. (2022). Physical and physiological characteristics of kickboxers: A systematic review. Arch. Budo.

[2] DeWeese B.H., Hornsby G., Stone M., Stone M.H. (2015). The training process: Planning for strength–power training in track and field. Part 1: Theoretical aspects. J. Sport Health Sci..

[3] Cimadoro G. (2017). Acute neuromuscular, cognitive and physiological responses to a Japanese kickboxing competition in semi-professional fighters. J. Sports Med. Phys. Fit..

[4] Pavelka R., Třebický V., Třebická Fialová J., Zdobinský A., Coufalová K., Havlíček J., Tufano J.J. (2020). Acute fatigue affects reaction times and reaction consistency in Mixed Martial Arts fighters. PLoS ONE.

[5] Turner A.N. (2009). Strength and conditioning for Muay Thai athletes. Strength Cond. J..

[6] Salci Y. (2015). The metabolic demands and ability to sustain work outputs during kickboxing competitions. Int. J. Perform. Anal. Sport.

[7] Slimani M., Chaabene H., Miarka B., Chamari K. (2017). The activity profile of elite low-kick kickboxing competition. Int. J. Sports Physiol. Perform..

[8] Ouergui I., Davis P., Houcine N., Marzouki H., Zaouali M., Franchini E., Gmada N., Bouhlel E. (2016). Hormonal, physiological, and physical performance during simulated kickboxing combat: Differences between winners and losers. Int. J. Sports Physiol. Perform..

[9] Slimani M., Miarka B., Briki W., Cheour F. (2016). Comparison of mental toughness and power test performances in high-level kickboxers by competitive success. Asian J. Sports Med..

[10] Ouergui I., Hammouda O., Chtourou H., Zarrouk N., Rebai H., Chaouachi A. (2013). Anaerobic upper and lower body power measurements and perception of fatigue during a kick boxing match. J. Sports Med. Phys. Fitness.

[11] Ouergui I., Hssin N., Haddad M., Franchini E., Behm D.G., Wong D.P., Gmada N., Bouhlel E. (2014). Time-motion analysis of elite male kickboxing competition. J. Strength Cond. Res..

[12] Cimadoro G., Mahaffey R., Babault N. (2019). Acute neuromuscular responses to short and long roundhouse kick striking paces in professional Muay Thai fighters. J. Sports Med. Phys. Fitness.

[13] Laett C.T., Silva R., Cossich C.F., Monteiro W., Barcellos L.C., Cossich V.R. (2023). Maximum and explosive strength in Brazilian kickboxing athletes: Asymmetries between limbs and the relationship with the single jump distance. Sport Sci. Health.

[14] Venegas-Carro M., Kramer A., Moreno-Villanueva M., Gruber M. (2022). Test–retest reliability and sensitivity of common strength and power tests over a period of 9 weeks. Sports.

[15] Hogarth L., Deakin G., Sinclair W. (2013). Are plyometric push-ups a reliable power assessment tool?. J. Aust. Strength Cond..

[16] Seifert L., Davids K. (2016). Ecological dynamics: A theoretical framework for understanding sport performance, physical education and physical activity. Springer Proceedings in Complexity.

[17] Marković G., Dizdar D., Jukić I., Cardinale M. (2004). Reliability and factorial validity of squat and countermovement jump tests. J. Strength Cond. Res..

[18] Ulupinar S., Özbay S., Gençoğlu C. (2021). Counter movement jump and sport specific frequency speed of kick test to discriminate between elite and sub-elite kickboxers. Acta Gymnica.

[19] Souza A.A., Bottaro M., Rocha V.A., Lage V., Tufano J.J., Vieira A. (2020). Reliability and test-retest agreement of mechanical variables obtained during countermovement jump. Int. J. Exerc. Sci..

[20] Springham M., Singh N., Stewart P., Matthews J., Jones I., Norton-Sherwood C., May D., Salter J., Strudwick A.J., Shaw J.W. (2024). Countermovement jump and isometric strength test–retest reliability in English Premier League academy football players. Int. J. Sports Physiol. Perform..

[21] Gillen Z.M., Miramonti A.A., McKay B.D., Jenkins N.D.M., Leutzinger T.J., Cramer J.T. (2018). Reliability and sensitivity of the power push-up test for upper-body strength and power in 6–15-year-old male athletes. J. Strength Cond. Res..

[22] Parry G.N., Herrington L.C., Horsley I.G. (2020). The test–retest reliability of force plate–derived parameters of the countermovement push-up as a power assessment tool. J. Sport Rehabil..

[23] Fawcett M., DeBeliso M. (2014). The Validity and Reliability of Push-Ups as a Measure of Upper Body Strength for 11–12 Year-Old Females. J. Fitness Res..

[24] Adams M.M., Hatch S.A., Winsor E.G., Parmelee C. (2022). Development of a standard push-up scale for college-aged females. Int. J. Exerc. Sci..

[25] World Medical Association (2021). Declaration of Helsinki: Ethical Principles for Medical Research Involving Human Subjects. https://www.wma.net/what-we-do/medical-ethics/declaration-of-helsinki/.

[26] Wu P.P.Y., Sterkenburg N., Everett K., Chapman D.W., White N., Mengersen K. (2019). Predicting fatigue using countermovement jump force-time signatures: PCA can distinguish neuromuscular versus metabolic fatigue. PLoS ONE.

[27] Robineau J., Jouaux T., Lacroix M., Babault N. (2012). Neuromuscular fatigue induced by a 90-minute soccer game modeling. J. Strength Cond. Res..

[28] Kalc M., Puš K., Paravlic A., Urbanc J., Šimunič B. (2023). Diagnostic accuracy of tensiomyography parameters for monitoring peripheral neuromuscular fatigue. J. Electromyogr. Kinesiol..

[29] Mehta R.K., Rhee J. (2021). Revealing Sex Differences During Upper and Lower Extremity Neuromuscular Fatigue in Older Adults Through a Neuroergonomics Approach. Front. Neuroergon..

[30] Cormack S.J., Mooney M.G., Morgan W., McGuigan M.R. (2013). Influence of neuromuscular fatigue on accelerometer load in elite Australian football players. Int. J. Sports Physiol. Perform..

[31] Alba-Jiménez C., Moreno-Doutres D., Peña J. (2022). Trends assessing neuromuscular fatigue in team sports: A narrative review. Sports.

